# Comparison of health-related quality of life between patients with different metatarsalgia types and matched healthy controls: a cross-sectional analysis

**DOI:** 10.1590/1516-3180.0220190918

**Published:** 2018-10-22

**Authors:** Natalia Tovaruela-Carrión, Daniel López-López, Marta Elena Losa-Iglesias, Verónica Álvarez-Ruíz, Gemma Melero-González, César Calvo-Lobo, Ricardo Becerro-de Bengoa-Vallejo

**Affiliations:** I DP, MSc, PhD. Associate Professor, Department of Podiatry, Universidad de Sevilla, Sevilla, Spain.; II DP, BSc, MSc, PhD. Senior Professor and Researcher, Research, Health and Podiatry Unit, Department of Health Sciences, School of Nursing and Podiatry, Universidade da Coruña, Ferrol, Coruña, Spain.; III RN, DP, MSc, PhD. Full Professor, School of Health Sciences, Universidad Rey Juan Carlos, Madrid, Alcorcón, Spain.; IV DP, MSc, PhD. External Collaborator, Department of Podiatry, Universidad de Sevilla, Sevilla, Spain.; V DP, MSc, PhD. External Collaborator, Department of Podiatry, Universidad de Sevilla, Sevilla, Spain.; VI PT, MSc, PhD. Assistant Professor, Department of Nursing and Physical Therapy, School of Health Sciences, Universidad de León, Ponferrada, León, Spain.; VII RN, BSc, MLIS, DPM, DHL, PhD, FFPM RCPS. Full Professor, Universidad Complutense de Madrid, Madrid, Spain.

**Keywords:** Foot deformities, Foot diseases, Metatarsalgia, Quality of life

## Abstract

**BACKGROUND:**

Metatarsalgia can be considered to be a common complaint in clinical practice. The aim of this study was to compare quality of life (QoL) between participants with different metatarsalgia types and matched-paired healthy controls.

**DESIGN AND SETTING:**

A cross-sectional analysis on a sample of 124 participants of median age ± interquartile range of 55 ± 22 years was carried out in the University Clinic of Podiatric Medicine and Surgery, Ferrol, Spain. They presented primary (n = 31), secondary (n = 31) or iatrogenic (n = 31) metatarsalgia, or were matched-paired healthy controls (n = 31).

**METHODS:**

Self-reported domain scores were obtained using the Foot Health Status Questionnaire (FHSQ) and were compared between the participants with metatarsalgia and between these and the healthy controls.

**RESULTS:**

Statistically significant differences were shown in all FHSQ domains (P ≤ 0.001). Post-hoc analyses showed statistically significant differences (P < 0.05) between the metatarsalgia types in relation to the matched healthy control group, such that the participants with metatarsalgia presented impaired foot-specific and general health-related QoL (lower FHSQ scores).

**CONCLUSION:**

This study demonstrated that presence of metatarsalgia had a negative impact on foot health-related QoL. Foot-specific health and general health were poorer among patients with metatarsalgia, especially among those with secondary and iatrogenic metatarsalgia, in comparison with matched healthy controls.

## INTRODUCTION

One of the most common forms of soreness of the feet is metatarsalgia. It is defined as acute or chronic pain in one or more metatarsophalangeal joints.^1^^,^^2^^,^^3^^,^^4^^,^^5^^,^^6^ This condition may be characterized as one of the most frequent symptoms in subjects with foot problems. Its prevalence in the general population is 10% and this may increase up to 50%-95% in older adults. Metatarsalgia can be considered to be the most frequent cause of foot pain in middle-aged women, accounting for approximately 85% of foot pain in that population.^7^^,^^8^^,^^9^ Up to 80% of the population may develop some kind of soreness in the metatarsal region over their lifetimes.^10^ Prevalence of 10% was reported in one population, with predominance in females.^11^ Foot soreness may affect approximately one-third of community-dwelling older adults.^12^ In a prospective cohort study among adults aged 50 years and over, the incidence of disabling foot soreness was found to reach 8.1% after a three-year follow-up and it increased with increasing age.^13^

Forefoot pain or metatarsalgia is a frequent symptom secondary to various conditions. Thus, knowledge of anatomical pathology in this area needs to be improved in order to achieve better differentiation regarding these processes.^9^ Metatarsalgia may not be limited only to pain in the foot sole: it may also be dorsal, lateral or medial, or be in a combination of these three regions. The local soreness may be accompanied by hyperkeratosis, helomas, claw or hammer toe deformities or subluxation of the metatarsophalangeal joints, or may be attributed to iatrogenic surgery.^2^^,^^14^ Mechanical metatarsalgia produces pain in the forefoot accompanied by plantar hyperkeratosis because of the key role of the metatarsal heads.^15^

Regarding the pathophysiology and treatment of metatarsalgia, this condition should be understood as secondary to anatomical and biomechanical alterations.^15^ Its origins may be varied, and some causes (e.g. rheumatoid arthritis) may be very complex. A wide degree of variability regarding the possible causal factors of metatarsalgia has been reported. Nevertheless, gait and posture biomechanics, along with foot and ankle deformities, may be considered to be key factors.^16^^,^^17^ Metatarsalgia may be secondary to damage (mechanical or of other origin) to anatomical structures surrounding the joint (capsule, ligaments, vessels, bone, cartilage, nerves, tendons, bursa, subcutaneous tissue or skin). ^1^^,^^2^^,^^3^^,^^4^^,^^5^^,^^6^ Metatarsalgia may be secondary to three groups of etiological factors: general diseases (i.e. inflammatory, metabolic, neurological or congenital conditions); anatomical and functional alterations (i.e. mechanical, static or propulsion factors); and iatrogenic or traumatic factors.^18^ Local bone and joint deformities, metabolic conditions, neuropathies and autoimmune conditions seem to be associated with metatarsalgia. However, the most significant associations are probably bipedal biomechanical alterations, inappropriate footwear use, repeated trauma and non-walking habits.^9^

This major health problem seems to be more frequent among females (85% of the population affected). Gout or rheumatoid arthritis may generate metatarsalgia, and this involves the distal metatarsophalangeal and interphalangeal joints.^9^ The soreness may be located in the metatarsal region of the forefoot and may increase through plantar pressure during standing and walking. Biomechanical alterations secondary to use of inappropriate footwear or deformities (e.g. hallux rigidus) may produce metatarsalgia in which the pain seems to be transmitted laterally because of load-shifting from the hallux to the smaller toes.^19^ The point at which metatarsalgia is generated is a key factor in understanding its cause.

Various classifications for metatarsalgia have been described in the literature.^2^^,^^20^ Primary metatarsalgia consists of first-ray hypermobility or metatarsal plantar flexion, prominent metatarsal heads (i.e. due to arthritis, tumors, infection or congenital or hereditary conditions), metatarsal length discrepancy and equinus condition (i.e. high-arched feet with contracture of the triceps surae muscles). Secondary metatarsalgia consists of metabolic disorders (e.g. gout), systemic conditions (e.g. rheumatoid arthritis or metatarsal phalangeal joints), trauma, neurological conditions (e.g. Morton’s neuroma or tarsal tunnel syndrome) and Freiberg disease. Iatrogenic metatarsalgia results from failure of surgical treatment for hallux abducto valgus consisting of arthrodesis of the first metatarsophalangeal joint, metatarsal osteotomy and shortened second ray.

A risk of falls and a decrease in physical activity may appear secondarily to foot pain, and these may promote a reduction in the quality of life (QoL).^21^^,^^22^ Reduction in physical activity has been reported to increase mortality.^23^ Regular daily walking may be healthy and may lead to a longer life.^24^ In addition, use of inadequate footwear may be very frequent among older adults, and this is highly related to forefoot deformities such as hallux valgus or hammer or claw toes.^25^ An increase in plantar pressure under the metatarsal heads may occur secondarily to these deformities, thus generating metatarsalgia.^26^ The risk of falls was shown to be higher among adults with foot soreness or toe deformities.^27^^,^^28^ Therefore, early comprehensive podiatric intervention should be recommended in order to prevent falls among older adults with disabling foot soreness.^29^

We hypothesized that increasing degrees of severity of metatarsalgia will decrease the QoL of patients who suffer from this condition.

## OBJECTIVE

The aim of this study was to compare the impacts of various degrees of metatarsalgia on quality of life relating to foot health, in a sample of patients with metatarsalgia and in healthy control subjects.

## METHODS

### Design and sample

A cross-sectional analysis was carried out from September 2015 to April 2016 at a clinic of podiatric medicine and surgery in the city of Ferrol, in the province of A Coruña, Spain, to study quality of life among subjects with metatarsalgia. All consecutive patients with foot pain who were seen at this clinic during this period were invited to participate in this study. Non-probabilistic convenience sampling was used in order to recruit participants, and the ages of these subjects ranged from 20 to 87 years. All subjects were required to be able to walk independently without an assistive device.

The following subjects were excluded: people with immune-comprised treatment, neurological conditions, lack of autonomy in daily activities or cognitive impairment (as determined using the Short Portable Mental Status Questionnaire, with scores < 7); and participants who declined to sign the consent form.

#### Sample size calculation

The sample size calculation was carried out by means of the one-way analysis of variance F test (fixed effects-omnibus ANOVA) using the G*Power 3.1.9.2 software. It was based on the general health domain of the FHSQ^30^ of a pilot study (n = 60 participants) with four groups, taking the mean: 15 patients with primary metatarsalgia (72.00 points), 15 patients with secondary metatarsalgia (57.33 points), 15 patients with iatrogenic metatarsalgia (59.33 points) and 15 matched-paired healthy controls (79.33 points). The total standard deviation (SD) within each group was 25.86 points. An effect size of 0.35, an α error probability of 0.05 and a power (1-β error probability) of 0.90 were used for the sample size calculation. Therefore, the total size of the sample was determined to be a minimum of 120 participants, i.e. 30 for each group. In the end, a total sample of 124 participants, i.e. 21 per group, was included in this study.

### Procedures

A single podiatrist researcher carried out all measurements. Height and weight were measured and body mass index (BMI) was calculated. The degree of metatarsalgia was established through a foot examination that was conducted in accordance with the classification proposed by Espinosa et al. In this, three kinds of metatarsalgia (primary, secondary and iatrogenic) are defined by using the mini-Lachman test to evaluate the integrity of the toe plantar plate at the metatarsophalangeal joint.^20^

Primary metatarsalgia may be secondary to mild translation, signifying that the plantar plate is intact. Secondary metatarsalgia may appear when the plantar plate is torn or attenuated secondary to reducible dislocation of the joint that may occur when mild force is exerted.^31^ Iatrogenic metatarsalgia may be diagnosed as an occurrence secondary to the past medical history. Patients without metatarsalgia and showing integrity of the toe plantar plate at the metatarsophalangeal joint, without signs or symptoms related with this condition, were included as controls, following the classification proposed by Espinosa et al.^20^ These controls were recruited if they had sociodemographic characteristics that were similar to those of the case groups.

The study subjects self-reported their conditions using the Foot Health Status Questionnaire (FHSQ). This questionnaire regarding health-related QoL is intended specifically for the foot and has been recognized as a validated tool that has been translated into Spanish.^29^^,^^32^^,^^33^ Both foot-specific and general health-related QoL have been evaluated using the FHSQ (version 1.03),^33^ which consists of three main sections.

Section 1 of FHSQ consists of 13 items reflecting four foot health-related domains: foot pain; foot function; footwear; and general foot health. This section has shown a high degree of content, criterion and construct validity (Cronbach α = 0.89-0.95) and high retest reliability (intraclass correlation coefficient = 0.74-0.92)^22^ and has been determined to be the most appropriate measurement of health-related QoL for patients with chronic plantar heel pain.^34^ Each domain has a specific number of questions, of which four refer to pain, four to function, three to footwear and two to general foot health. The pain and function evaluations are based on physical phenomena. Footwear assessment includes practical issues relating to shoe availability and comfort, and the perception of general foot health is based on patients’ self-assessment of the state of their feet. Several possible answers are presented on a Likert-type ordinal scale. The scale descriptors vary for each domain, and the participant determines only one response as the most appropriate. The questionnaire does not provide any overall score but, rather, it generates a score for each domain. The responses are analyzed using computer software (FHSQ, version 1.03) and the scores range from 0 to 100. A score of 0 represents the worst health-related QoL state for the foot and 100 indicates the best possible health-related QoL state for the foot. In addition, the software provides graphical illustrations of the outcomes.

Section 2 includes items that reflect four general health-related domains: general health, physical activity, social capacity and vigor. The domains and items in this section are largely adapted from the short form-36 (SF-36) survey,^34^ which has been validated (the Cronbach α ranges from 0.89 to 0.95), with high retest reliability (the intraclass correlation coefficient ranges from 0.74 to 0.92) for the Spanish version.^29^^,^^35^

Section 3 collects data on socioeconomic status, comorbidities, service utilization, satisfaction and medical records.

### Ethical considerations

The Research Ethics Committee of the University of Coruña, Spain, approved this study, under registration number C.E.I. 01/2015, with application date March 6, 2015. All the patients participated voluntarily and gave their consent in written form. The ethical standards for human research and the Declaration of Helsinki (World Medical Association) and rules from other appropriate national/institutional organizations were respected.

### Statistical analysis

Descriptive analysis on the variables included in the study and comparisons between patients with primary, secondary and iatrogenic metatarsalgia and between these and matched healthy controls were made in accordance with the sample size calculation. The Shapiro-Wilk test was performed to determine the distribution of the variables. Categorical data appeared as frequencies and percentages and between-group comparisons were analyzed using the chi-square (χ^2^) test. Parametric quantitative data were analyzed using means and standard deviations (SD) and the range (maximum and minimum values). Between-group comparisons were analyzed using one-way analysis of variance (ANOVA). Non-parametric quantitative data, including medians, interquartile ranges (IR) and ranges (maximum and minimum values), along with between-group comparisons, were analyzed using the Kruskal-Wallis test. Because all FHSQ domains presented nonparametric data, the Kruskal-Wallis test was complemented by means of the Wilcoxon test, with adjustment using Bonferroni’s correction in order to determine any post-hoc differences. The IBM SPSS 22.0 statistics package was used for the analyses on the data. FHSQ version 1.03 was used to obtain QoL scores relating to foot health. In all the analyses, P < 0.05 (with a 95% confidence interval) was considered statistically significant, unless otherwise stated.

## RESULTS

A total sample of 124 people between 20 and 87 years of age completed the study. The sample included 76 women (61.3%) and 48 men (38.7%). Table 1 shows that the patients’ clinical and sociodemographic characteristics were homogenous, given that there were no statistically significant differences (P > 0.05).

**Table 1.** Sociodemographic characteristics of patients with primary, secondary and iatrogenic metatarsalgia and matched healthy controls

Sociodemographic characteristics		Control group n = 31	Primary metatarsalgia n = 31	Secondary metatarsalgia n = 31	Iatrogenic metatarsalgia n = 31	P-value
Age (years)		41.00 ± 30.00 (20-80)	58.00 ± 15.00 (30-65)	59.00 ± 14.00 (20-82)	55.00 ± 12.00 (25-87)	0.051^†^
Weight (kg)		72.00 ± 12.00 (56-100)	72.00 ± 17.00 (50-110)	74.00 ± 18.00 (50-110)	72.00 ± 11.00 (58-100)	0.656^†^
Height (cm)		169.71 ± 6.62 (155-180)	166.97 ± 7.96 (151-182)	168.29 ± 8.89 (150-183)	166.35 ± 7.51 (155-190)	0.338*
BMI (kg/cm^2^)		24.70 ± 3.20 (20-80)	25.00 ± 5.70 (20-44)	25.70 ± 6.60 (19-39)	24.70 ± 3.20 (19-41)	0.439^†^
Sex	Male	14 (11.3%)	9 (7.3%)	14 (11.3%)	11 (8.9%)	0.485^‡^
	Female	17 (13.7%)	22 (17.7%)	17 (13.7%)	20 (16.1%)	
Professional activity	Student	2 (1.6%)	0 (0%)	1 (0.8%)	1 (0.8%)	0.276^‡^
	Freelance	4 (3.2%)	1 (0.8%)	2 (1.6%)	1 (0.8%)	
	Employed	14 (11.3%)	12 (9.7%)	6 (4.8%)	12 (9.7%)	
	Unemployed	3 (2.4%)	11 (8.9%)	11 (8.9%)	9 (7.3%)	
	Retired	8 (6.5%)	7 (5.6%)	11 (8.9)	8 (6.5%)	
Study level	I. primary	5 (4%)	8 (6.5%)	6 (6.5%)	6 (4.8%)	0.912^‡^
	C. primary	6 (4.8%)	7 (5.6%)	8 (6.5%)	7 (5.6%)	
	Secondary	8 (6.5%)	11 (8.9%)	8 (6.5%)	9 (7.3%)	
	Degree	9 (7.3%)	4 (3.2%)	5 (4%)	7 (5.6%)	
	H. Degree	3 (2.4%)	1 (0.8%)	4 (3.2%)	2 (1.6%)	
Civil status	Single	3 (2.4%)	2 (1.6%)	2 (1.6%)	1 (0.8%)	0.089^‡^
	Divorced	2 (1.6%)	3 (4.4%)	1 (0.8%)	4 (5.2%)	
	Widowed	2 (1.6%)	2 (1.6%)	5 (4%)	7 (5.6%)	
	Couple	4 (3.2%)	1 (0.8%)	1 (0.8%)	7 (5.6%)	
	Married	20 (16.1%)	23 (18.5%)	22 (17.7%)	12 (9.7%)	

BMI = body mass index; C = complete; I = incomplete; H = higher. *Mean ± standard deviation (SD), range (min-max) and one-way analysis of variance (ANOVA) were used. ^†^Median ± interquartile range (IR), range (min-max) and Kruskal-Wallis test were used. ^‡^Frequency, percentage (%) and chi-square test (χ^2^) were used.

In the control group, there were 31 study participants. In the metatarsalgia group, there were 93 study participants, with 31 participants in each group (primary, secondary and iatrogenic metatarsalgia). The results from comparisons between the FHSQ scores according to the degree of metatarsalgia and between these groups and matched controls are shown in Table 2. Section One of the FHSQ showed statistically significant differences (P < 0.001) for the four foot-specific domains:

1. pain;
2. function;
3. health; and
4. footwear.

**Table 2.** Comparisons of FHSQ scores between patients with primary, secondary and iatrogenic metatarsalgia and matched healthy controls

	Control (C) group Median (IR) n =31	Primary (P) metatarsalgia group Median (IR) n = 31	Secondary (S) metatarsalgia group Median (IR) n = 31	Iatrogenic (I) metatarsalgia group Median (IR) n = 31	Kruskal-Wallis P-value	Paired group comparison Wilcoxon P-value
Foot pain	90.62 ± 21.88 (29.38-100)	72.50 ± 39.38 (10.63-100)	43.75 ± 47.50 (0-100)	54.37 ± 56.25 (0-100)	< 0.001	1. P vs C** 2. S vs C^†^ 3. I vs C^†^ 4. S vs P 5. I vs P 6. I vs S
Foot function	100.0 ± 12.50 (25-100)	81.25 ± 43.75 (6.25-100)	56.25 ± 50.00 (0-100)	68.75 ± 56.25 (0-100)	< 0.001	1. P vs C 2. S vs C^†^ 3. I vs C** 4. S vs P 5. I vs P 6. I vs S
Footwear	75.00 ± 50.00 (25-100)	50.00 ± 50.00 (0-100)	50.00 ± 33.33 (0-100)	50.00 ± 41.67 (0-100)	< 0.001	1. P vs C* 2. S vs C** 3. I vs C** 4. S vs P 5. I vs P 6. I vs S
General foot health	79.67 ± 20.00 (25-100)	60.00 ± 60.00 (12.50-100)	42.50 ± 35.00 (0-100)	85.00 ± 20.00 (25-100)	< 0.001	1. P vs C* 2. S vs C^†^ 3. I vs C** 4. S vs P 5. I vs P 6. I vs S
General health	100.0 ± 30.00 (40-100)	80.00 ± 40.00 (10-100)	50.00 ± 40.00 (0-100)	70.00 ± 40.00 (0-100)	< 0.001	1. P vs C 2. S vs C^†^ 3. I vs C^†^ 4. S vs P 5. I vs P 6. I vs S
Physical activity	100.0 ± 11.11 (38.89-100)	88.88 ± 33.33 (38.89-100)	66.66 ± 27.78 (11.11-100)	83.33 ± 33.33 (5.56-100)	0.001	1. P vs C 2. S vs C^†^ 3. I vs C* 4. S vs P 5. I vs P 6. I vs S
Social capacity	100.0 ± 00.00 (50-100)	87.50 ± 25.00 (37.50-100)	75.00 ± 50.00 (25-100)	87.50 ± 50.00 (12.50-100)	0.001	1. P vs C 2. S vs C** 3. I vs C* 4. S vs P 5. I vs P 6. I vs S
Vigor	75.00 ± 25.00 (50-100)	62.50 ± 31.25 (12.50-100)	50.00 ± 18.75 (25-100)	56.25 ± 18.75 (25-100)	< 0.001	1. P vs C 2. S vs C^†^ 3. I vs C* 4. S vs P 5. I vs P 6. I vs S

FHSQ = Foot Health Status Questionnaire Survey; IR = interquartile range; vs = versus. *Statistically significant at P-value < 0.05 adjusted using Bonferroni’s correction. **Statistically significant at P-value < 0.01 adjusted using Bonferroni’s correction. ^†^Statistically significant at P-value < 0.001 adjusted using Bonferroni’s correction.

Post-hoc analyses showed statistically significant differences (P < 0.05) between all metatarsalgia types with regard to the matched healthy control group, for foot pain and footwear; and also between the secondary and iatrogenic metatarsalgia types with regard to the matched healthy control group for foot function and general foot health, thus showing impaired foot-specific health-related quality of life (lower FHSQ scores).

Section Two of the FHSQ provided statistically significant differences (P ≤ 0.001) for the four overall wellbeing domains:

1. overall health;
2. physical function;
3. social capacity; and
4. vigor.

Post-hoc analyses showed statistically significant differences (P < 0.05) between the secondary and iatrogenic metatarsalgia types with regard to the matched healthy control group, thus showing impaired general health-related quality of life (lower FHSQ scores).

The rest of the comparisons did not show any statistically significant differences (P > 0.05).

## DISCUSSION

The main aim of this study was to assess the impact of different degrees of metatarsalgia on health-related QoL in a sample of patients with such conditions, compared with a healthy control group. Proper foot health may be essential, and 75% of adults complain of foot soreness in association with significant foot problems, with evidence of arthritic changes on X-rays.^36^ Falls may be common among older adults, with consequences such as major threats to their health, along with higher costs and economic burdens for healthcare services. Foot problems have been identified as risk factors for falls, and painful feet may be associated with increased risk of falls and decreased mobility and QoL.^37^ Despite this, the role of physical rehabilitation in fall prevention programs may be negligible,^38^ and supporting evidence for this practice in relation to metatarsalgia is scarce. Menz and Lord reported that older adults with foot pain and plantar hyperkeratosis showed worse balance and functional ability than those with other foot problems and no soreness.^39^

Numerous studies have addressed the issue of metatarsalgia, regarding its clinical features and treatment.^40^^,^^41^^,^^42^ Nevertheless, there has been no research evaluating the impact of metatarsalgia on patients’ QoL. Gines et al. assessed QoL among patients with hallux valgus and, additionally, compared these findings with results from patients who suffered from hallux valgus and metatarsalgia in order to state which of these two groups showed worse QoL.^42^ Because of the lack of epidemiological data regarding metatarsalgia, it is difficult to estimate the influence that this condition has on foot health-related QoL among participants with different kinds of metatarsalgia. The results from the present study confirmed that subjects with metatarsalgia presented lower scores in all dimensions relating to footwear, general foot health, foot pain, foot function, vigor, physical activity, social capacity and general health, in comparison with the healthy control group. From a physical rehabilitation point of view, it is important to understand the results from this study in order to develop healthcare programs for promoting foot health according to the degree of these patients’ metatarsalgia.

We did not find any reports in the literature evaluating the impact of metatarsalgia on QoL. It would be beneficial to establish the extent to which metatarsalgia may affect general health and foot health. This would be useful prior to formulating a physical rehabilitation program, in order to determine the effectiveness of physical rehabilitation programs and the benefits that are obtained secondarily to them. In this manner, the impact on QoL, from before to after the program, can be compared. Painful feet may limit functional ability and mobility among patients, and rehabilitation nurses need to be aware of these factors in order to develop rehabilitation programs. Soreness secondary to metatarsalgia may be reduced through debridement using a scalpel, which may additionally improve functional ability and should be considered to be a key factor in implementing a multidisciplinary approach for fall prevention.

Podiatrists, physical therapists and physicians, in collaboration with a multidisciplinary rehabilitative nursing program, may develop an environment in which knowledge about foot mechanics and footwear can be improved, so as to prevent improper conditions and improve patients’ health-related QoL.

Regarding metatarsal pain management, physical rehabilitation may teach foot pain management skills and help patients to achieve better adherence to treatment and acquire more effective coping mechanisms. In this manner, the negative effects from foot pain can be minimized. Thus, nurses may have the responsibility to manage foot care and improve patients’ QoL.

The impact of our results may be difficult to compare with that of other studies due to differences regarding criteria and methodological variations. To the authors’ knowledge, there are no other reports in the literature regarding QoL and foot health among participants with different degrees of metatarsalgia. Nevertheless, there are various limitations to the present study that need to be acknowledged. First, a larger sample size and greater diversity of subjects from different countries would be beneficial for strengthening this study. In addition, this would help to identify differences relating to different cultures and the mechanisms involved. This highlights the need for additional studies in order to define rehabilitative nursing interventions that might improve patients’ foot health-related QoL.

## CONCLUSIONS

Among people suffering from metatarsalgia, their condition had a negative impact on foot health-related QoL. There were significant reductions in foot-specific and general health among patients with metatarsalgia, especially regarding secondary and iatrogenic metatarsalgia, in relation to matched healthy controls.

The findings presented here have important consequences for proper rehabilitative nursing care, control over foot conditions and prevention of the appearance or development of metatarsalgia, as key factors in the process of monitoring foot functionality.
